# A Highly Sensitive ppb-Level H_2_ Gas Sensor Based on Pt/PtO and Pd/PdO*_x_* Co-Decorated WO_3_ Nanofibers Prepared by Electrospinning

**DOI:** 10.3390/s26103079

**Published:** 2026-05-13

**Authors:** Zhipeng Tang, Jinshun Wang, Lixin Zhang, Qiuxia Li, Chen Yang, Yuhao Pang, Yingying Yang, Jingwei Chen, Qingkuan Meng, Qiang Jing

**Affiliations:** Laboratory of Functional Molecules and Materials, School of Physics and Optoelectronic Engineering, Shandong University of Technology, 266 Xincun Xi Road, Zibo 255000, China; 15166901551@163.com (Z.T.); 19546304631@163.com (J.W.); zzhanglixin7@163.com (L.Z.); 19862540463@163.com (Q.L.); yc2092694103@163.com (C.Y.); ppyh123gg@163.com (Y.P.); yangyingying@sdut.edu.cn (Y.Y.); jingqiang@sdut.edu.cn (Q.J.)

**Keywords:** resistive H_2_ gas sensor, WO_3_-based gas sensor, noble metal Pt, noble metal Pd, bimetallic decoration, electrospun nanofibers

## Abstract

A highly sensitive ppb-level resistive H_2_ gas sensor was fabricated based on Pt/PtO and Pd/PdO*_x_* co-decorated WO_3_ nanofibers prepared via electrospinning and calcination. The optimized sensor based on 2 at% Pt–2 at% Pd co-decorated WO_3_ nanofibers exhibited reliable detection toward 100 ppb H_2_ at an optimized operating temperature of 170 °C. Upon 2 at% Pd decoration, the response of the WO_3_-based sensor increased from 1, corresponding to almost no response, to 55 (R_*a*_/R_*g*_) toward 100 ppm H_2_. Further introduction of 2 at% Pt reduced the optimal operating temperature of the 2 at% Pd-decorated WO_3_-based sensor from 200 °C to 170 °C and enhanced the response by approximately twofold. The optimal sensor exhibits excellent linear response characteristics, high selectivity, good response repeatability, and long-term operational stability. The enhanced sensing performance is attributed to the catalytic capability and possible spillover-related effects of Pd/PdO*_x_* and Pt/PtO toward H_2_/O_2_, as well as depletion-layer modulation induced by the heterostructures between Pt/PtO and WO_3_, and Pd/PdO*_x_* and WO_3_. These synergistic catalytic and electronic sensitization effects collectively contribute to the high sensitivity toward H_2_. These results indicate that the proposed resistive H_2_ sensor holds significant potential for practical hydrogen-sensing applications.

## 1. Introduction

Hydrogen ($H_{2}$) is widely regarded as a promising clean and sustainable energy carrier due to its exceptionally high gravimetric energy density and the absence of carbon emissions during combustion [1,2,3]. With the rapid expansion of hydrogen-based technologies in areas such as energy storage, transportation, and industrial applications, the need for dependable and efficient hydrogen monitoring systems has grown considerably [4,5,6,7,8,9,10,11]. Nevertheless, hydrogen exhibits unique physicochemical properties that distinguish it from conventional gases [1,3,12]. Its extremely small molecular size and high diffusion coefficient facilitate rapid leakage and dispersion, and its broad flammability range in air (4–75 vol%) presents significant safety hazards even at low concentrations [1,3,13,14,15]. As a result, the development of hydrogen sensors with high sensitivity, selectivity, and long-term stability for trace-level $H_{2}$ detection is critically important for both industrial safety and the practical deployment of hydrogen energy systems [7,8,16,17].

Among the various sensing materials explored, metal oxide semiconductors (MOS) have attracted extensive attention because of their simple device architecture, low fabrication cost, and compatibility with miniaturized sensor platforms [18,19,20,21,22]. Tungsten trioxide (${\mathrm{WO}}_{3}$), an n-type semiconductor, has emerged as a particularly promising hydrogen-sensing material owing to its favorable band structure, excellent chemical stability, and strong interaction with reducing gases [18,23,24,25]. It has been well established that the sensing mechanism of ${\mathrm{WO}}_{3}$ is primarily governed by surface adsorption and desorption processes involving ionized oxygen species, which regulate the surface depletion layer and, consequently, the electrical resistance of the material [26,27]. Therefore, the gas-sensing performance of ${\mathrm{WO}}_{3}$-based sensors is strongly influenced by factors such as morphology, specific surface area, and charge transport pathways [18,23,27]. One-dimensional nanostructures, including nanofibers, nanowires, and nanotubes, offer distinct advantages for gas-sensing applications. Their high surface-to-volume ratio and continuous electron conduction channels promote efficient gas diffusion and rapid surface reactions, leading to improved sensitivity and faster response–recovery characteristics [20,28,29,30,31]. Electrospinning is widely recognized as a versatile and effective approach for producing one-dimensional metal oxide nanofibers with tunable diameters, porosity, and film uniformity. Electrospun ${\mathrm{WO}}_{3}$ nanofiber networks typically form highly porous sensing layers, which are especially advantageous for detecting gases at low concentrations [32,33,34]. Despite these merits, pristine ${\mathrm{WO}}_{3}$-based sensors often exhibit relatively high optimal operating temperatures and insufficient selectivity, limiting their practical applicability [35,36].

To overcome these drawbacks, noble metal functionalization has been extensively employed as an effective strategy to improve gas-sensing performance [10,11,35,37,38,39,40,41]. In particular, platinum (Pt), palladium (Pd) and their oxides have drawn considerable interest due to their outstanding catalytic activity toward hydrogen adsorption, dissociation, spillover, and interfacial charge modulation [38,39,42,43,44]. Pt is also known to facilitate oxygen activation and its spillover processes [38,44,45]. Beyond single-metal (and its oxides) modification, the incorporation of bimetallic Pt–Pd species (and their oxides) provides additional opportunities for synergistic enhancement of hydrogen-sensing performance [10,39,40,46]. Compared with monometallic systems, Pt–Pd bimetallic configurations (and their oxides) can concurrently optimize oxygen activation, hydrogen dissociation, and interfacial charge transfer as a result of their complementary catalytic and electronic properties [39,42,46,47,48,49]. In such hybrid systems, the formation of $\mathrm{Pt}/\mathrm{Pd}-{\mathrm{WO}}_{3}$ Schottky junctions and oxide/semiconductor heterointerfaces gives rise to pronounced space-charge regions at the metal–semiconductor and semiconductor–semiconductor interfaces [47,49,50]. These interfacial depletion layers are highly responsive to surface chemical reactions, enabling amplified modulation of electrical resistance upon hydrogen exposure [51,52]. In this context, rational surface decoration of nanofiber architectures can introduce additional catalytic or charge-transfer sites and thereby improve gas adsorption, reaction kinetics, and sensing stability. However, compared with VOC sensing systems, the development of bimetallic noble-metal-decorated WO_3_ nanofibers for ppb-level H_2_ detection remains relatively limited.

In this work, Pt–Pd (and their oxides) co-decorated ${\mathrm{WO}}_{3}$ nanofibers were fabricated via electrospinning followed by controlled calcination. A series of Pt–Pd (and their oxides) co-decorated ${\mathrm{WO}}_{3}$ nanofibers with different decoration ratios were synthesized to optimize the co-decorating ratio of Pt and Pd (and their oxides). The hydrogen-sensing performance of the sensors based on these nanofibers was evaluated over a broad concentration range down to the ppb level. The sensor exhibits a low detection limit of 100 ppb at the optimal operating temperature of 170 °C. The good linear response, selectivity and response repeatability were also observed. This study provides a rational design strategy for high-performance ${\mathrm{WO}}_{3}$-based H_2_ sensors and provides valuable insights into the synergistic effects of bimetallic sensitization for trace H_2_ detection.

## 2. Experimental Section

### 2.1. Preparation of the Sensing Materials via Electrospinning

**Figure 1:**
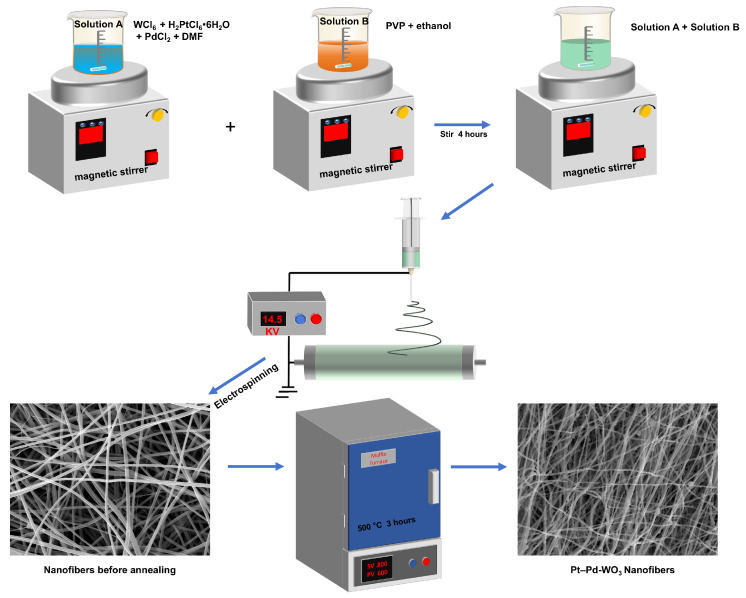
Schematic illustration of the synthesis process of the sensing materials. The precursor nanofibers exhibit diameters of approximately 170–300 nm before calcination, while the diameters of the calcined WO_3_-based nanofibers decrease to approximately 100–170 nm, as determined from the SEM observations.

As illustrated in Figure 1, pure ${\mathrm{WO}}_{3}$ nanofibers and Pt–Pd bimetallic–decorated ${\mathrm{WO}}_{3}$ nanofibers with different decoration ratios were successfully synthesized via electrospinning. Briefly, 1.5 g of tungsten chloride (${\mathrm{WCl}}_{6}$) was dissolved in 10 g of N,N-dimethylformamide (DMF) together with predetermined amounts of $H_{2}{\mathrm{PtCl}}_{6}\;\cdot \;6H_{2}O$ and ${\mathrm{PdCl}}_{2}$. The masses of the metal precursors were adjusted according to the target Pt/Pd decoration ratios. Specifically, Pd-decorated WO_3_ nanofibers with Pd:W target molar ratios of 1:100, 2:100, and 3:100 were first prepared and denoted as 1 at% Pd–WO_3_, 2 at% Pd–WO_3_, and 3 at% Pd–WO_3_, respectively. Based on the optimization of the Pd decorating ratio, Pt–Pd co-decorated WO_3_ nanofibers were further prepared by fixing the Pd content at 2 at% and varying the Pt content from 1 to 3 at%. The corresponding Pt:Pd:W target molar ratios were 1:2:100, 2:2:100, and 3:2:100, and the samples were denoted as 1 at% Pt–2 at% Pd–WO_3_, 2 at% Pt–2 at% Pd–WO_3_, and 3 at% Pt–2 at% Pd–WO_3_, respectively. Among them, the 2 at% Pt–2 at% Pd-decorated WO_3_ sample was denoted as Sample B. These samples were used for material characterization and gas-sensing performance evaluation. The resulting solution was magnetically stirred at room temperature for 4 h to ensure complete dissolution and homogeneous dispersion of the metal salts. This precursor-incorporation strategy offers several advantages over post-decoration methods such as impregnation, drop-casting, or surface deposition. Since the Pt and Pd precursors are introduced directly into the electrospinning precursor solution, the metal species can be more uniformly distributed throughout the nanofiber precursor during fiber formation. After calcination, this process promotes intimate contact between Pt/Pd-derived species and the WO_3_ matrix, which is beneficial for forming abundant noble-metal/WO_3_ and metal-oxide/WO_3_ interfacial sites. In addition, the one-step incorporation of Pt and Pd during electrospinning avoids repeated post-treatment steps and helps preserve the continuous porous nanofiber network. These features are favorable for gas diffusion, catalytic activation, and interfacial charge-transfer processes in resistive H_2_ sensing.

In parallel, 2.4 g of polyvinylpyrrolidone (PVP, molecular weight ≈1,300,000 g mol^−1^) was dissolved in 10 mL of anhydrous ethanol and stirred for 4 h to obtain a homogeneous polymer solution [32,34]. The two solutions were then mixed and stirred for an additional 4 h to form a homogeneous and viscous electrospinning precursor. Although no quantitative rheological measurement was performed, the precursor solution showed suitable viscosity for electrospinning, as evidenced by the formation of a stable Taylor cone and continuous bead-free nanofibers during the electrospinning process. The prepared precursor solution was loaded into a 10 mL syringe and electrospun through a metal needle at a constant flow rate of 0.1 mL/h. During electrospinning, the distance between the needle tip and the rotating collector was fixed at 20 cm. A positive voltage of +11.5 kV was applied to the needle, while a negative voltage of –3 kV was applied to the collector. Under the applied electric field, continuous fibers were ejected and uniformly deposited onto an aluminum foil–covered collector, forming a dense and continuous nanofiber membrane.

The as-spun nanofibers were subsequently calcined in a muffle furnace under an air atmosphere. The temperature was increased at a rate of 1 °C min^−1^ to 500 °C and maintained for 3 h. This calcination process ensured complete removal of the polymer matrix and promoted the crystallization of ${\mathrm{WO}}_{3}$ and the incorporated Pt and Pd species, yielding well-crystallized pure ${\mathrm{WO}}_{3}$ and Pt–Pd co-decorated ${\mathrm{WO}}_{3}$ nanofibers with dense and robust structures.

### 2.2. Fabrication of the Resistive H_2_ Gas Sensor Device

The calcined nanofiber material was gently ground in an agate mortar to fragment large pieces and improve its dispersion. During this process, the nanofibers were not completely destroyed, but were mainly shortened into fibrous fragments, which helped retain the one-dimensional morphology and the porous network structure of the sensing material. The calcined nanofibers were then dispersed in an appropriate amount of anhydrous ethanol to form a homogeneous slurry. The slurry was uniformly applied to the Al_2_O_3_ substrate using a clean brush, ensuring that the sensing material was evenly distributed over the surface. After deposition, the sensor was dried at 120 °C for 1 h to remove residual ethanol and stabilize the sensing layer. Finally, the fabricated resistive H_2_ gas sensor device was mounted in a gas-sensing measurement system for performance evaluation.

### 2.3. Material Characterization

The crystal structure and phase purity of pristine WO_3_ and Pt–Pd-decorated WO_3_ were characterized by X-ray diffraction (XRD; SmartLab, Rigaku Corporation, Tokyo, Japan) using Cu K_*α*_ radiation. X-ray photoelectron spectroscopy (XPS; ESCALAB 250Xi, Thermo Scientific, Waltham, MA, USA) was employed to analyze the chemical states of Pd, Pt, W, and O. The surface morphology of WO_3_ was examined by scanning electron microscopy (SEM; Quanta 250 FEG, FEI, Hillsboro, OR, USA). High-resolution transmission electron microscopy (HRTEM; Tecnai G2 F20, FEI, Hillsboro, OR, USA) was used to observe the lattice fringes.

### 2.4. Gas Sensing Measurement

**Figure 2:**
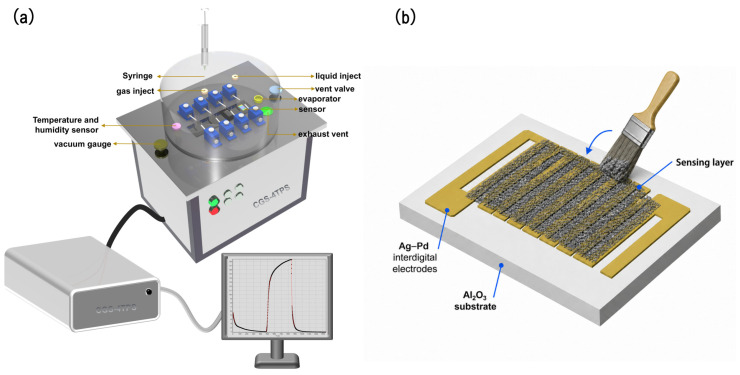
(**a**) Schematic illustration of the instrumentation used for gas-sensing performance measurements. (**b**) Schematic illustration of the slurry coating process, showing the application of the sensing layer to the Al_2_O_3_ substrate using a clean brush to ensure uniform distribution.

A custom-designed static-mode intelligent gas-sensing analysis system (CGS-4TPS, Beijing Elite Technology Co., Ltd., Beijing, China) was used to assess the gas-sensing performance of the fabricated sensors. The system is equipped with four independent channels and integrates temperature and pressure control capabilities. Figure 2 presents a schematic diagram of the experimental setup. Precise temperature regulation is achievable from room temperature up to 500 °C, with an accuracy of ±1 °C. Electrical signals were acquired by contacting the electrode surface with two probes. Prior to measurements, all sensors were preheated to the preset operating temperature. To determine the optimal working temperature, the sensor response toward 100 ppm H_2_ was measured at a series of preset operating temperatures after the baseline resistance became stable at each temperature. The temperature at which the sensor exhibited the highest response together with stable response–recovery behavior was defined as the optimal working temperature. After the sensor resistance reached a stable baseline, a predetermined amount of target gas was injected into the test chamber through a rubber septum using a gas-tight microsyringe. For the ppb-level H_2_ measurements, the target gas was obtained using a static volumetric dilution method. A certified H_2_ standard gas with a known concentration was used as the source gas and diluted with dry air in the sealed test chamber. To reduce the error caused by injecting an extremely small gas volume, stepwise dilution was performed before the final target concentration was obtained. The target H_2_ concentration was determined according to the dilution relationship between the certified concentration of the H_2_ standard gas, the calibrated volume of the sealed test chamber, and the injected gas volume measured by the gas-tight microsyringe:

$$C_{\mathrm{target}}=C_{\mathrm{standard}}\times \frac{V_{\mathrm{inj}}}{V_{s}},$$

where $C_{\mathrm{target}}$ is the target H_2_ concentration, $C_{\mathrm{standard}}$ is the certified concentration of the H_2_ standard gas, $V_{\mathrm{inj}}$ is the injected gas volume, and $V_{s}$ is the calibrated volume of the sealed test chamber. Before each sensing test, sufficient diffusion time was allowed to ensure that the injected H_2_ was uniformly distributed in the chamber. Following each measurement, the chamber was opened to allow the sensor to recover to its initial baseline in air. During the measurements, ambient humidity, sensor operating temperature, and resistance changes were recorded simultaneously. The sensor response was defined as $S=R_{a}/R_{g}$, where $R_{a}$ and $R_{g}$ correspond to the sensor resistance in air and in the target gas, respectively. The response time was defined as the time required for the sensor resistance to reach 90% of the total resistance change after exposure to the target gas. The recovery time was defined as the time required for the sensor resistance to recover 90% of the total resistance change after the target gas was removed and the sensor was re-exposed to air. No mathematical baseline correction or smoothing was applied to the original resistance curves. The baseline resistance $R_{a}$ was obtained from the stable resistance value in air immediately before exposure to the target gas, and $R_{g}$ was taken as the resistance value after the sensor response reached a steady state in the target gas. For repeatability analysis, repeated response–recovery cycles were quantified using the relative standard deviation (RSD) of the response values:

$$\mathrm{RSD}=\frac{s}{\bar{S}}\times 100\%,$$

where $\bar{S}$ and *s* are the mean value and standard deviation of the response values obtained from repeated sensing cycles, respectively. The limit of detection (LOD) was estimated based on the signal-to-noise ratio criterion. The baseline noise was obtained from the standard deviation (σ) of the stable resistance signal recorded in air and converted into response units. The LOD was calculated according to

$$\mathrm{LOD}=\frac{3\sigma }{k},$$

where *k* is the slope of the calibration curve in the low-concentration region. A signal-to-noise ratio of 3 was used as the criterion for reliable detection. Among the fabricated sensors described in Section 2.1, the sensor fabricated using Sample B exhibited the best gas-sensing performance and was denoted as Sensor B. Subsequent performance evaluations were primarily conducted using this sensor.

## 3. Results and Discussion

### 3.1. Basic Characteristics of the Sensing Materials

**Figure 3:**
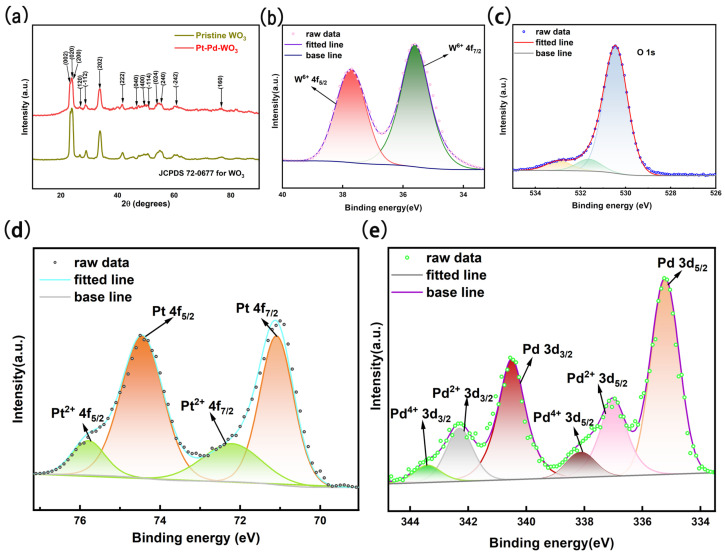
(**a**) XRD patterns of pristine WO_3_ and Pt–Pd-decorated WO_3_ nanofibers. (**b**–**e**) High-resolution core-level spectra of W 4f, O 1s, Pt 4f, and Pd 3d for the Pt–Pd–WO_3_ sample with the best gas-sensing performance (Sample B), respectively.

The crystal structures of pristine WO_3_ and the optimized 2 at% Pt–2 at% Pd-decorated WO_3_ sample (Sample B) were investigated by X-ray diffraction (XRD), as shown in Figure 3a. Both samples exhibit nearly identical diffraction patterns, which can be well indexed to the monoclinic WO_3_ phase (JCPDS No. 72-0677). No distinct diffraction peaks corresponding to PtO*_x_* or PdO_*y*_ species are observed in Sample B, which can be reasonably attributed to the low loading amounts of Pt and Pd and/or their high dispersion and ultrasmall particle size within the WO_3_ matrix. To further elucidate the surface chemical states of the sensing material with the best gas sensing performance, X-ray photoelectron spectroscopy (XPS) was carried out. Figure 3b presents the core-level spectra of W 4f. The peaks at binding energies of 35.6 and 37.74 eV are assigned to W^6+^, corresponding to the oxidizing state of WO_3_ [53]. As shown in Figure 3c, the O 1s spectrum can be deconvoluted into three distinct components centered at approximately 530.4, 531.5, and 532.9 eV [54,55], corresponding to lattice oxygen (O*_L_*), defective oxygen (O*_V_*), and chemisorbed/deionized oxygen (O*_C_*), respectively. O*_L_* is generally regarded as an inactive oxygen species that does not participate in sensing reactions with the target gas. In contrast, O*_V_* is associated with oxygen vacancies and interstitials, which facilitate gas adsorption and desorption processes. O*_C_* corresponds to oxygen species (O^2−^, O^−^, and $O_{2}^{-}$) chemisorbed on the surface of the sensing material, which can directly react with target gases and induce electron transfer [45,56,57]. The XPS peak area ratio is $S_{O_{L}}$:$S_{O_{V}}$:$S_{O_{C}}$ = 12:1.5:1. The Pt 4f spectrum (Figure 3d) can be deconvoluted into two chemical states, corresponding to metallic Pt^0^ and oxidized Pt^2+^ species. The doublet located at 71.1 and 74.5 eV is assigned to Pt^0^ (Pt 4f_7/2_ and 4f_5/2_), whereas the peaks at 72.5 and 75.7 eV are attributed to Pt^2+^ species, indicating the presence of partially oxidized platinum on the surface [55,57,58]. The XPS peak area ratio is $S_{{\mathrm{Pt}}^{0}}$:$S_{{\mathrm{Pt}}^{2+}}$ = 5:2. Similarly, the Pd 3d spectrum (Figure 3e) exhibits three pairs of spin–orbit components. The Pd 3d_5/2_ and 3d_3/2_ peaks centered at 335.2 eV and 340.5 eV, 336.6 eV and 341.9 eV, and 338.1 and 343.4 eV correspond to Pd^0^, Pd^2+^, and Pd^4+^ species, respectively [55,59,60], confirming the coexistence of metallic and oxidized palladium states. The XPS peak area ratio is $S_{{\mathrm{Pd}}^{0}}$:$S_{{\mathrm{Pd}}^{2+}}$:$S_{{\mathrm{Pd}}^{4+}}$ = 8:3:1.

**Figure 4:**
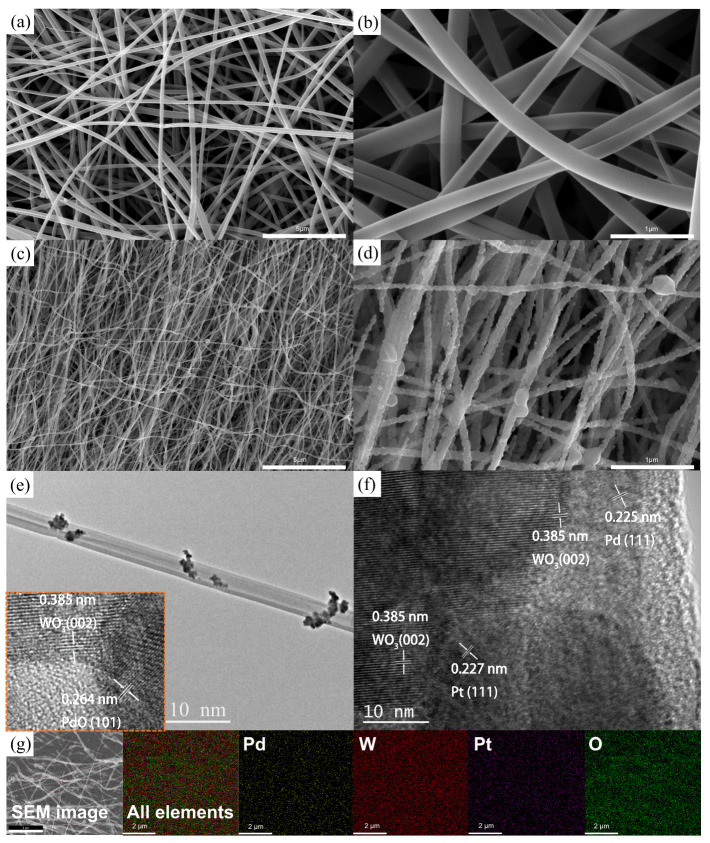
(**a**,**b**) SEM images of the precursor nanofibers before calcination at low and high magnifications, respectively. (**c**,**d**) SEM images of the nanofibers after calcination at the corresponding magnifications. (**e**,**f**) TEM and HRTEM images of the Pt–Pd-decorated WO_3_ nanofibers, respectively. (**g**) EDS elemental mapping of the Pt–Pd–WO_3_ nanofibers as sensing materials.The detailed and enlarged version of the EDS mapping is provided in the Supporting Information as Figure S9.

Figure 4a,b present SEM images of the precursor nanofibers before calcination at low and high magnifications, respectively. The nanofibers exhibit diameters in the range of 170–300 nm. As observed, the fiber surfaces are smooth and uniform, and no obvious structural defects are detected. Figure 4c,d display SEM images of the nanofibers after calcination at low and high magnifications, respectively. Following calcination, the fiber diameters decrease to approximately 100–170 nm, which can be attributed to the removal of organic components and structural densification. In addition, small crystalline features are clearly observed on the surface of the WO_3_ nanofibers, indicating successful crystallization. Figure 4e,f show the TEM and HRTEM images of Sample B. As shown in Figure 4e, the nanofibers maintain a uniform fibrous morphology with an average diameter of approximately 130 nm. Discrete nanoparticles on the fiber surface are attributed to the decorated Pt–Pd species. The inset HRTEM image in Figure 4e reveals lattice fringes with an interplanar spacing of 0.264 nm, which can be indexed to the PdO (101) plane, confirming the oxidized state of Pd species. The HRTEM image in Figure 4f further shows clear lattice spacings of 0.227 and 0.225 nm, corresponding to the (111) planes of Pt (JCPDS No. 4-802) and Pd (JCPDS No. 46-1043), respectively, and a spacing of 0.385 nm assigned to the (002) plane of WO_3_ (JCPDS No. 72-0677). The spatial distribution of the constituent elements was further investigated by energy-dispersive X-ray spectroscopy (EDS). As shown in Figure 4g, both Pt and Pd are uniformly distributed across the WO_3_ nanofibers. Such homogeneous dispersion maximizes the exposure of active catalytic sites and facilitates efficient interfacial contact between the noble metal oxides and the WO_3_ matrix. While platinum and palladium oxides provide catalytically active sites for gas adsorption and surface reactions, the WO_3_ framework serves as a robust scaffold that preserves the fibrous architecture during repeated redox processes [33]. The synergistic integration of structural stability and surface functionality underpins the reliable gas-sensing and catalytic performance of the Pt–Pd–WO_3_ nanofiber system [34].

### 3.2. Gas-Sensing Performance

**Figure 5:**
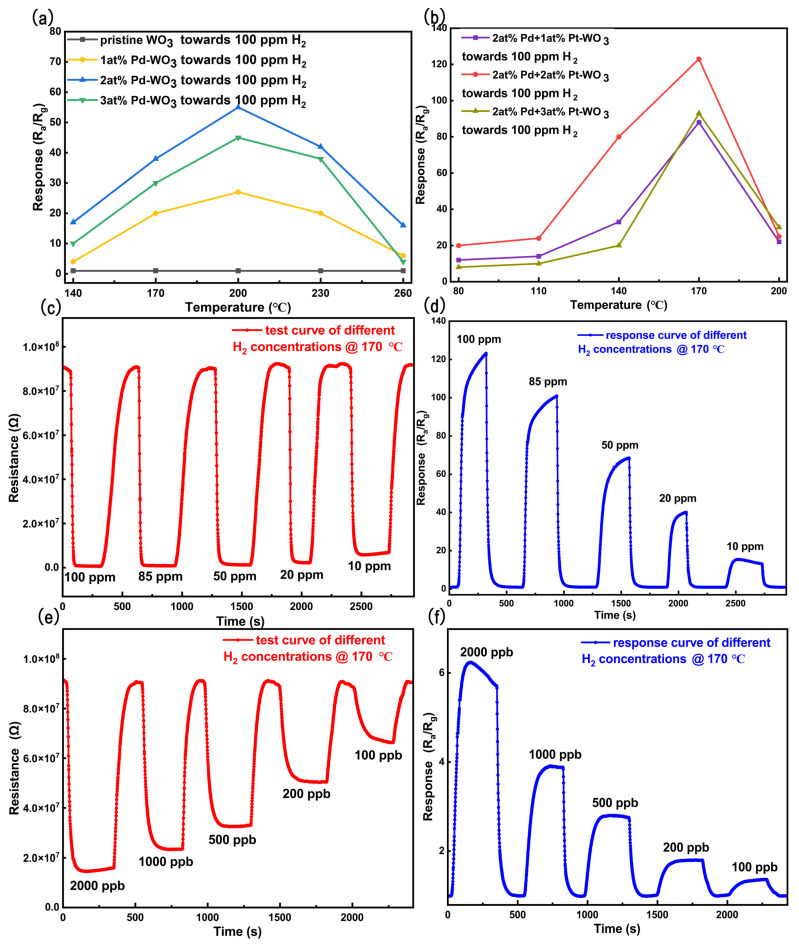
(**a**) Temperature-dependent response values of the sensors with different Pd decorating ratios toward 100 ppm H_2_. (**b**) Temperature-dependent response values of sensors with different Pt decorating ratios at a fixed Pd decorating ratio of 2 at%. (**c**) Original resistance variation curves of the sensor based on 2 at% Pt–2 at% Pd-decorated WO_3_ toward H_2_ concentrations ranging from 10 to 100 ppm at 170 °C. (**d**) Corresponding response curves expressed as $R_{a}/R_{g}$. (**e**) Original resistance variation curves of the same sensor toward low H_2_ concentrations ranging from 100 to 2000 ppb at 170 °C. (**f**) Corresponding response curves expressed as $R_{a}/R_{g}$.

**Figure 6:**
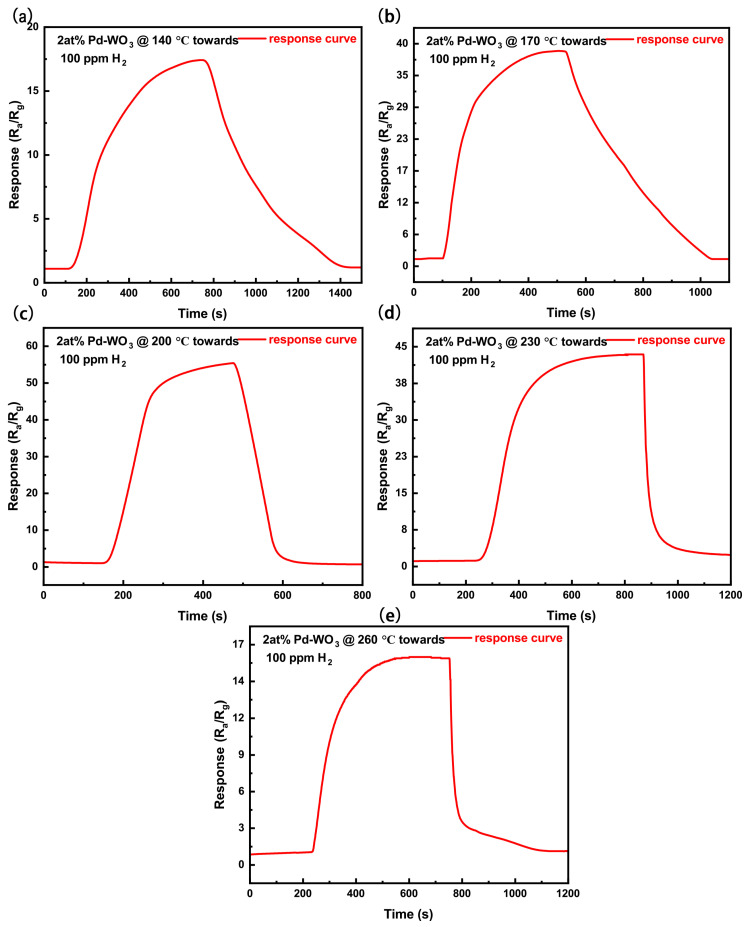
(**a**–**e**) Dynamic response curves of the sensor based on 2 at% Pd-decorated ${\mathrm{WO}}_{3}$ towards 100 ppm $H_{2}$, measured at operating temperatures of 140 °C, 170 °C, 200 °C, 230 °C, and 260 °C, respectively.

**Figure 7:**
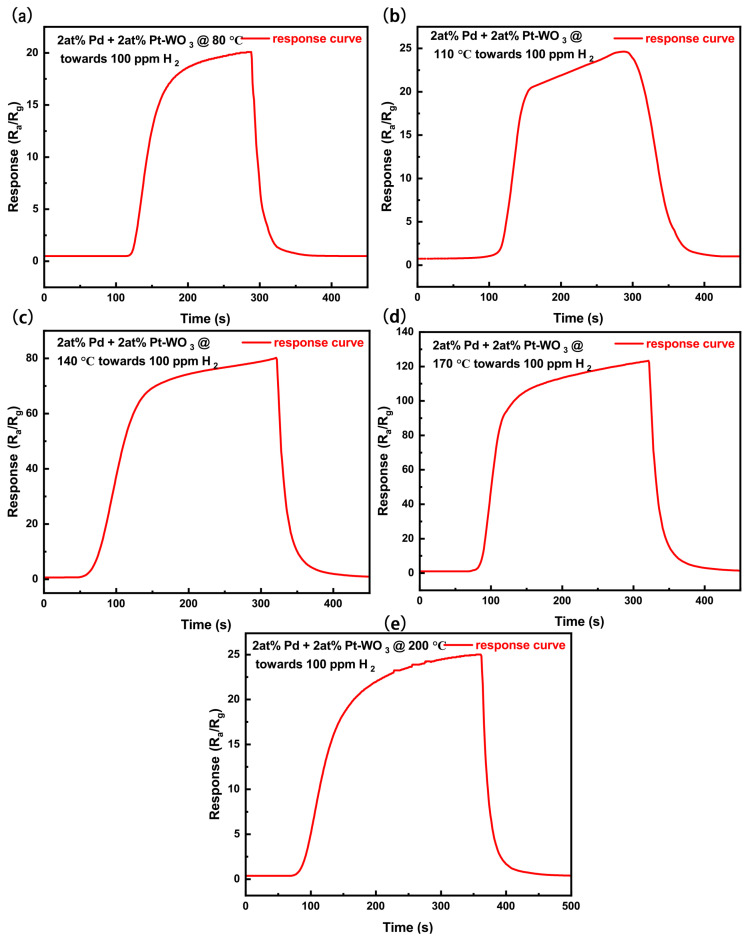
(**a**–**e**) Dynamic response curves of the sensor based on 2 at% Pt–2 at% Pd-decorated ${\mathrm{WO}}_{3}$ toward 100 ppm $H_{2}$, measured at operating temperatures of 80 °C, 110 °C, 140 °C, 170 °C, and 200 °C, respectively.

To determine the optimal decorating ratios of Pt and Pd, a series of systematic experiments were conducted to optimize the Pt–Pd decoration composition. Since the sensing performance of metal–oxide–semiconductor (MOS) gas sensors is highly dependent on the operating temperature, identifying an optimal working temperature is essential for achieving reliable sensing performance [61]. Figure 5a presents the temperature-dependent responses of sensors with different Pd decorating ratios. As shown, the sensor decorated with 2 at% Pd exhibits the best gas-sensing performance, with an optimal operating temperature of 200 °C. Figure 6 displays the response curves of the 2 at% Pd-decorated WO_3_ sensor toward 100 ppm H_2_, measured at various operating temperatures (140–260 °C). Given that the optimal Pd decorating ratio was determined to be 2 at%, further optimization was performed by varying the Pt decorating ratio while maintaining the Pd content at 2 at%. Figure 5b shows the temperature-dependent responses of sensors with a fixed Pd decorating ratio of 2 at% and different Pt decorating ratios. According to the temperature-dependent response results in Figure 5b, the sensor fabricated using Sample B exhibits the highest response toward 100 ppm H_2_ at 170 °C. Therefore, 170 °C was determined as the optimal working temperature of Sensor B. Specifically, the response of the 2 at% Pd–2 at% Pt-decorated WO_3_ sensor reaches 123 (R_*a*_/R_*g*_) at 170 °C, compared with a response value of 55 (R_*a*_/R_*g*_) at 200 °C for the sensor decorated with 2 at% Pd only. These results indicate that Pt decoration not only significantly enhances the sensor response but also effectively lowers the optimal operating temperature. Figure 7 shows the response curves of the 2 at% Pt–2 at% Pd-decorated WO_3_ sensor toward 100 ppm H_2_, measured at different temperatures (80–200 °C). As shown in Figure 6 and Figure 7, and Figures S2–S7, temperature significantly influences the sensor’s response and recovery times. At lower temperatures, the response time is slower due to decreased molecular diffusion and reduced interactions at the sensor surface. Conversely, as the temperature increases, the response time decreases due to faster gas diffusion and adsorption kinetics. However, the recovery time also shows a faster recovery at higher temperatures due to enhanced desorption of gas molecules. This demonstrates the importance of optimizing the working temperature to achieve both fast response and stable recovery, which are crucial for practical gas sensing applications. The dynamic sensing responses of Sensor B toward H_2_ over a concentration range of 10–100 ppm at the optimal operating temperature of 170 °C are presented in Figure 5c,d, which show the resistance variation curves and the corresponding response curves (R_*a*_/R_*g*_), respectively. Figure 5e,f further present the dynamic response behavior of Sensor B in terms of resistance variation and R_*a*_/R_*g*_ values toward H_2_ over a concentration range of 100–2000 ppb at 170 °C.

**Figure 8:**
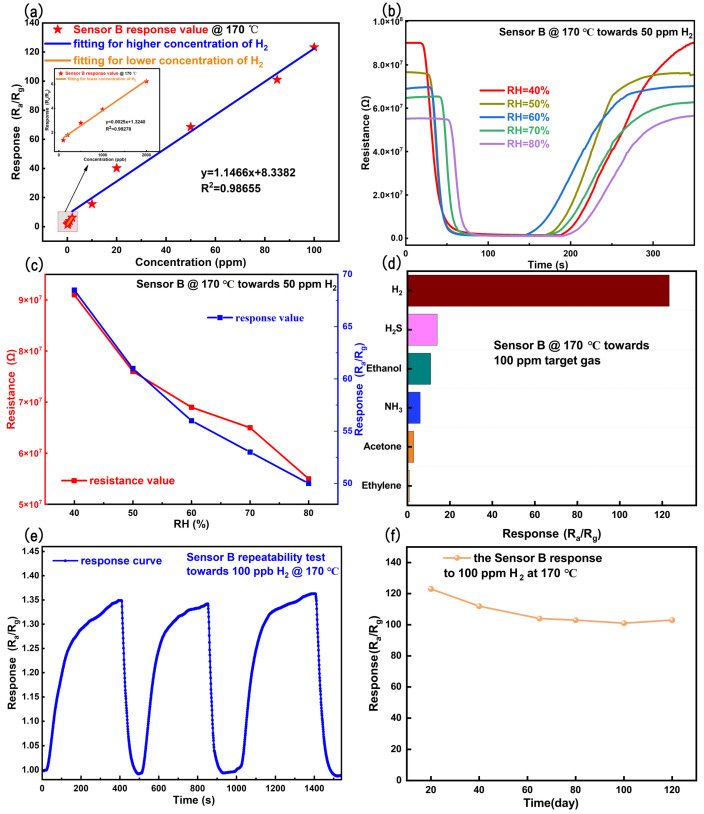
(**a**) Linear fitting of the concentration-dependent response values; the inset highlights the fitting results in the low $H_{2}$ concentration range. (**b**) Original response curves of the sensor toward 50 ppm $H_{2}$ under different relative humidity (RH) conditions ranging from 40% to 80%. (**c**) RH-dependent baseline resistance and corresponding response values. (**d**) Selectivity evaluation of the sensor. (**e**) Response repeatability test of the sensor. (**f**) Long-term stability test of the sensor.

Figure 8a illustrates the linear fitting of the concentration-dependent response of Sensor B toward H_2_ over a broad concentration range from 100 ppb to 100 ppm at the optimal operating temperature of 170 °C. Taking 2 ppm as the inflection point, the sensing behavior can be divided into two linear regions: 100 ppb–2 ppm and 2–100 ppm. In the low-concentration region (100 ppb–2 ppm), a pronounced linear relationship is observed, with a slope of 0.0025 ppb^−1^ and a correlation coefficient of 0.99278. The linear fitting can be expressed as Y=0.0025x+1.3240, where *Y* denotes the sensor response and *x* represents the H_2_ concentration. To rigorously evaluate the detection capability, the signal-to-noise ratio and LOD were further calculated from the stable air-baseline signal. The baseline noise was converted into response units, and the conservative noise level was estimated to be $\sigma =1.20\times 10^{-3}$. Based on the slope of the low-concentration calibration curve (k=0.0025 ppb^−1^), the theoretical LOD was calculated as

$$\mathrm{LOD}=\frac{3\sigma }{k}=\frac{3\times 1.20\times 10^{-3}}{0.0025}\approx 1.4\;\mathrm{ppb}.$$

In addition, the average response of Sensor B toward 100 ppb H_2_ was calculated to be 1.358, corresponding to a response change of ΔS=0.358. The signal-to-noise ratio was therefore estimated to be

$$S/N=\frac{\Delta S}{\sigma }=\frac{0.358}{1.20\times 10^{-3}}\approx 298,$$

which is much higher than the criterion of 3. These results confirm that 100 ppb H_2_ can be reliably detected by Sensor B under the present experimental conditions. In the higher concentration range (2–100 ppm), the sensor response likewise exhibits a linear dependence on H_2_ concentration, yielding a slope of 1.1466 ppm^−1^ and a correlation coefficient of 0.98655, described by Y=1.1466x+8.3382. The influence of relative humidity (RH) on the sensor response was further investigated. Figure 8b shows the response curves of the sensor toward 50 ppm H_2_ under different RH conditions in the range of 40%–80%. Figure 8c presents the RH-dependent baseline resistance and response values. It is observed that both the baseline resistance and response decrease with increasing RH. The gradual decrease in baseline resistance with increasing relative humidity can be attributed to the interaction between H_2_O molecules and the WO_3_ surface. As an n-type metal–oxide semiconductor, WO_3_ exhibits electron depletion in dry air due to the adsorption of ionized oxygen species. With increasing humidity, adsorbed H_2_O molecules partially dissociate on the surface to form hydroxyl groups, which consume surface oxygen species and release trapped electrons back into the conduction band. Consequently, the surface depletion layer narrows and the grain-boundary potential barriers are reduced, leading to a decrease in baseline resistance [62,63,64,65,66]. Humidity exerts a detrimental effect on the sensing response, commonly referred to as the water-vapor poisoning effect [63,65,67]. When H_2_O molecules are adsorbed onto the sensing layer, they dissociate to form OH^−^. The possible surface reactions under humid conditions can be described as follows:

$$H_{2}O_{\left(\mathrm{gas}\right)}+O_{\left(\mathrm{ads}\right)}^{-}\to 2{\mathrm{OH}}_{\left(\mathrm{ads}\right)}^{-},$$

$$H_{2}O_{\left(\mathrm{gas}\right)}+O_{2(\mathrm{ads})}^{-}\to 2{\mathrm{OH}}_{\left(\mathrm{ads}\right)}^{-}+O_{\left(\mathrm{ads}\right)}^{-}.$$

These reactions indicate that adsorbed water molecules can consume reactive oxygen species and generate surface hydroxyl groups. The occupation of surface active sites by OH^−^ species suppresses the chemisorption of oxygen species and hinders the surface diffusion of target gas molecules, thereby degrading the sensing performance [63,65,67]. This humidity-dependent behavior should be considered in practical H_2_ sensing applications, because metal-oxide-based chemiresistive sensors are often operated in practical atmospheres at their optimized working temperatures, where variations in environmental humidity can affect surface adsorption, oxygen-species activation, charge-transfer processes, and gas diffusion. Previous studies on metal-oxide nanowire gas sensors have also emphasized that operating temperature and humidity are important factors influencing the sensing response and recovery behavior [68]. Therefore, the decrease in response under high-RH conditions observed in Figure 8b,c indicates that humidity calibration or compensation may be required for practical hydrogen leakage monitoring at the optimized operating temperature of Sensor B. Nevertheless, Sensor B still maintains a measurable response toward 50 ppm H_2_ even at 80% RH and 170 °C, suggesting its potential applicability under humid operating conditions. The gas selectivity of Sensor B was evaluated at 170 °C by comparing its responses toward 100 ppm H_2_ and several representative interfering gases, including H_2_S, ethanol, NH_3_, acetone, and ethylene, as shown in Figure 8d. All interfering gases were tested at the same concentration of 100 ppm to ensure a direct and fair comparison of gas selectivity. The selection of these interfering gases was based on practical H_2_ monitoring scenarios, such as hydrogen production, storage, transportation, chemical processing, and fuel-cell-related applications. In these environments, H_2_ sensors may be exposed to toxic or reducing gases, volatile organic compounds, and light hydrocarbons. Therefore, H_2_S and NH_3_ were selected as representative toxic/reducing gases, ethanol and acetone as representative volatile organic compounds, and ethylene as a representative light hydrocarbon. As shown in Figure 8d, Sensor B exhibits a markedly higher response to H_2_ than to these interfering gases, indicating its good selectivity toward hydrogen under practically relevant interference conditions. Comparable studies on Pt/Pd-modified WO_3_ and related hydrogen-sensing platforms also highlight that catalytic noble-metal loading and heterointerface engineering are crucial for balancing sensitivity, selectivity, and operating temperature [41,48,49,69,70,71,72,73,74,75,76,77,78,79,80,81,82,83,84]. Figure 8e illustrates the response repeatability of Sensor B toward 100 ppb H_2_. The response values obtained from repeated response–recovery cycles were used to calculate the RSD according to the method described in Section 2.4. The average response toward 100 ppb H_2_ was calculated to be approximately 1.36, with an RSD of less than 1.0%, confirming good repeatability at the lowest experimentally tested concentration. Although quantitative RSD analysis was performed for the repeated 100 ppb H_2_ cycles, the dynamic response curves over wider concentration ranges, including 100–2000 ppb and 10–100 ppm H_2_, show reversible response–recovery behavior at different concentrations. These results qualitatively support the reproducible sensing behavior of Sensor B over a broader concentration range. Figure 8f presents the long-term stability of Sensor B, indicating that the sensing response remains stable over a period of 120 days. Although no obvious response degradation was observed during the test period, possible degradation mechanisms should still be considered for long-term practical operation. For Pt/PtO- and Pd/PdO*_x_*-co-decorated WO_3_ nanofiber sensors, performance degradation may arise from several factors, such as the gradual aggregation or sintering of noble-metal nanoparticles during continuous operation at the optimized working temperature, changes in the oxidation states of Pt/PtO and Pd/PdO*_x_* during repeated H_2_/air exposure, partial formation and decomposition of PdH*_x_* species during hydrogen sensing cycles, and the adsorption of moisture or residual gas impurities on surface active sites. In addition, changes in oxygen vacancies and chemisorbed oxygen species on the WO_3_ surface may also affect the surface depletion layer and charge-transfer process, leading to response drift over extended operation. Therefore, further optimization of noble-metal dispersion, surface protection, and humidity calibration or compensation may be useful strategies for improving the long-term reliability of this type of hydrogen sensor.

The response and recovery times were determined according to the 90% resistance-change criterion described in Section 2.4. Table 1 presents a performance comparison between Sensor B and other representative Pt/Pd-modified WO_3_ hydrogen sensors. From a comprehensive performance perspective, Sensor B exhibits highly competitive H_2_-sensing performance, particularly in terms of low-concentration detection, moderate operating temperature, response repeatability, and long-term stability. As summarized in Table 1, many previously reported Pt- or Pd-modified WO_3_ sensors were mainly evaluated at relatively high H_2_ concentrations, typically in the ppm range. In contrast, the present Pt–Pd–WO_3_ nanofiber sensor can experimentally detect H_2_ down to 100 ppb at 170 °C. Although some reported sensors exhibit very large responses at high H_2_ concentrations, direct comparison based only on response magnitude is not sufficient because the tested concentration, operating temperature, response definition, and material morphology are different. From the viewpoint of practical early-stage hydrogen leakage warning, the ability to detect ppb-level H_2_ with stable response–recovery behavior is particularly important. Moreover, Sensor B shows good repeatability at 100 ppb H_2_ with an RSD below 1.0% and maintains stable performance over 120 days. Therefore, compared with representative Pt/Pd-modified WO_3_ sensors, the present sensor demonstrates clear advantages in trace-level H_2_ detection and long-term operational reliability.

### 3.3. Gas Sensing Mechanism

The decoration of Pt/PtO and Pd/PdO*_x_* on WO_3_ nanofibers enhances the gas sensing performance through synergistic effects of chemical (catalytic) sensitization and electronic sensitization.

#### 3.3.1. Catalytic Sensitization

The catalytic sensitization process can be described by the following surface reactions:

(1) $$O_{2}\left(\mathrm{gas}\right)\to O_{2(\mathrm{ads})},$$

(2) $$O_{2(\mathrm{ads})}+e^{-}↔O_{2(\mathrm{ads})}^{-},$$

(3) $$O_{2(\mathrm{ads})}^{-}+e^{-}↔2O_{\left(\mathrm{ads}\right)}^{-},$$

(4) $$H_{2}\left(\mathrm{gas}\right)\to 2H_{\left(\mathrm{atom}\right)},$$

(5) $$2H+O_{\left(\mathrm{ads}\right)}^{-}\to H_{2}O+e^{-},$$

(6) $$2H+O_{2(\mathrm{ads})}^{-}\to H_{2}O+2e^{-}.$$

**Figure 9:**
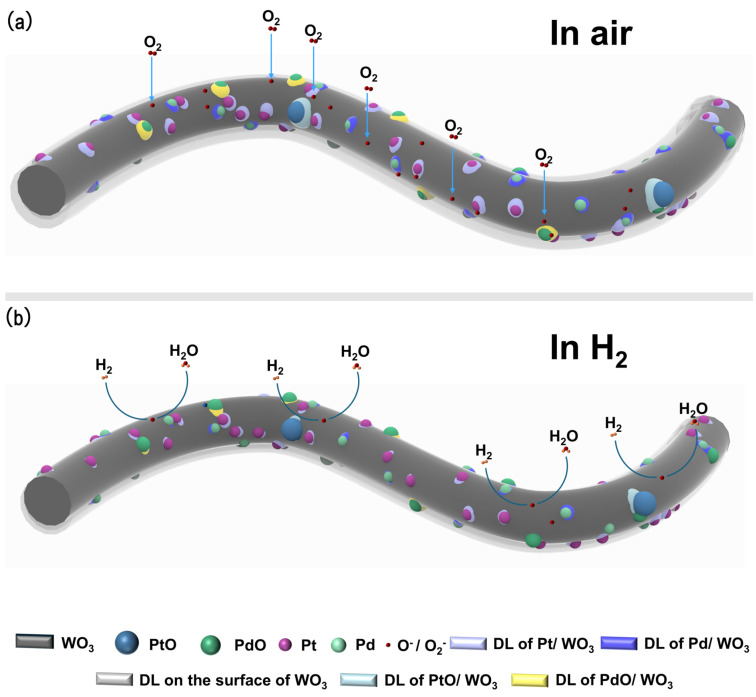
Schematic illustration of the hydrogen-sensing mechanism of the sensor based on Pt–Pd–WO_3_ nanofibers under air (**a**) and H_2_ atmospheres (**b**).

Upon exposure to air, oxygen molecules are first adsorbed onto the sensor surface, as described in Equation (1). The adsorbed oxygen molecules then extract electrons from the conduction band of WO_3_ to form ionized oxygen species such as $O_{2}^{-}$ and O^−^, as shown in Equations (2) and (3) [85,86,87]. As illustrated in Figure 9a, this electron-transfer process induces the formation of an electron depletion layer near the WO_3_ surface, leading to an increase in the surface resistance. When the sensor is exposed to H_2_, Pt/Pd sites facilitate the dissociation of H_2_ into atomic hydrogen, as shown in Equation (4). The generated hydrogen atoms subsequently react with pre-adsorbed oxygen species, as described in Equations (5) and (6), releasing electrons back into the conduction band of WO_3_ and resulting in a marked decrease in sensor resistance.

The introduction of Pt/Pd nanoparticles significantly alters the oxygen adsorption behavior. These noble metal sites provide energetically favorable locations for oxygen adsorption and activation. Owing to their superior catalytic activity relative to pristine WO_3_, oxygen molecules are more likely to interact with Pt/Pd surfaces, where they are transformed into highly reactive ionosorbed oxygen species. Subsequently, these activated species can migrate from the noble metal sites onto the WO_3_ surface via a spillover process, thereby increasing the overall density of reactive oxygen species involved in gas sensing reactions [88]. This effect is widely considered to be a dominant factor responsible for the improved sensing response and reduced operating temperature observed in noble-metal-modified chemiresistive gas sensors [88].

This catalytic sensitization mechanism is further supported by the XPS results discussed in Section 3.1. The O 1s spectrum confirms the coexistence of lattice oxygen, oxygen-vacancy-related oxygen, and chemisorbed oxygen species on the surface of Sample B. The oxygen-vacancy-related oxygen species indicate the presence of surface defects, which can provide favorable adsorption and activation sites for oxygen molecules. Meanwhile, the chemisorbed oxygen species can directly participate in the redox reaction with H_2_, thereby contributing to the resistance modulation during sensing. In addition, the Pt 4f and Pd 3d spectra reveal the coexistence of metallic Pt^0^/Pd^0^ and oxidized Pt^2+^/Pd^2+^/Pd^4+^ species. Metallic Pt and Pd can promote H_2_ adsorption and dissociation, whereas PtO*_x_* and PdO*_x_* can contribute to oxygen activation and the formation of metal-oxide/WO_3_ heterointerfaces. Therefore, the XPS results provide direct evidence for the coexistence of catalytic active sites, reactive oxygen species, and interfacial electronic structures, which collectively support the enhanced H_2_-sensing mechanism of the Pt/PtO- and Pd/PdO*_x_*-co-decorated WO_3_ nanofibers. In addition to oxygen activation, Pt/PtO and Pd/PdO*_x_* modification also enhances the interaction between the sensor surface and hydrogen molecules. Pt/Pd provides highly active sites that facilitate the dissociation of H_2_ into atomic hydrogen (H_2_ → 2H) (Equation (4)), effectively lowering the activation energy required for surface reactions [89,90]. The generated hydrogen atoms can migrate from the noble metal sites to the WO_3_ surface, where they react with pre-adsorbed oxygen species ($O_{2}^{-}$ or $O^{-}$). This reaction releases electrons back into the conduction band of WO_3_, resulting in a marked decrease in resistance and an enhanced sensing signal [90]. Consequently, the presence of Pt/Pd significantly improves the sensitivity, accelerates the response kinetics, and enables operation at lower temperatures compared with unmodified WO_3_ [89,91].

#### 3.3.2. Electronic Sensitization

**Figure 10:**
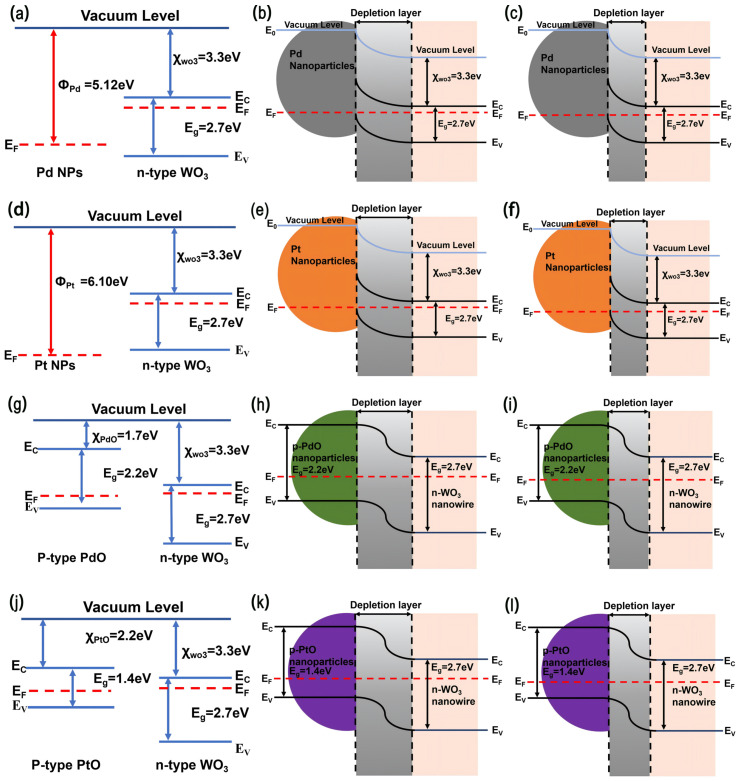
Schematic energy-band diagrams illustrating the interfacial electronic modulation mechanism of the Pt–Pd–WO_3_ nanofiber sensor. (**a**–**c**) Energy-band diagrams of the Pd/WO_3_ interface before contact, after contact in air, and after exposure to H_2_, respectively. (**d**–**l**) The same scenario for the Pt/WO_3_ interface, p-PdO/n-WO_3_ heterojunction and the p-PtO/n-WO_3_ heterojunction, respectively.

Beyond catalytic effects, Pt/Pd decoration also induces pronounced electronic sensitization through the formation of interfacial Schottky barriers at the metal/WO_3_ interface, as shown in Figure 10b,e. Due to the higher work function of Pt/Pd relative to WO_3_, electrons tend to transfer from WO_3_ to the noble metal, leading to the formation of a depletion region at the interface [92,93]. In addition, PtO*_x_* and PdO*_x_*, which typically exhibit p-type semiconductor behavior, can form p–n heterojunctions when coupled with n-type WO_3_. The formation of such heterojunctions further enlarges the depletion region at the interface (Figure 9a and Figure 10h,k), thereby increasing the baseline resistance of the sensor. This elevated resistance enhances the relative variation in resistance upon exposure to hydrogen. When H_2_ is introduced, the interfacial charge distribution is significantly modulated, resulting in an amplified sensing signal. Such interfacial electronic effects are widely recognized as key contributors to the improved performance of noble-metal-decorated WO_3_ sensors [87,92,93,94].

As described in Equations (5) and (6), exposure to H_2_ leads to reactions between dissociated hydrogen atoms and adsorbed oxygen species ($O_{2}^{-}$ and O^−^), producing H_2_O and releasing electrons back to the WO_3_ surface. This electron reinjection reduces the depletion layer width (as shown in Figure 9b and Figure 10c,f,i,l) and consequently decreases the sensor resistance.

In addition to these mechanisms, metallic Pd contributes to sensing enhancement through the formation of palladium hydride (PdH*_x_*) [95,96]. Due to its strong affinity for hydrogen, Pd can readily absorb hydrogen atoms following dissociative adsorption, leading to the formation of PdH*_x_* [95]. The generation of PdH*_x_* reduces the work function of Pd and consequently modifies the Schottky barrier at the Pd/WO_3_ interface [97]. This facilitates electron transfer into WO_3_, resulting in enhanced modulation of the depletion layer and improved sensing response [97]. Thus, PdH*_x_* introduces an additional electronic sensitization mechanism beyond conventional catalytic effects. Furthermore, the reversible transition between Pd and PdH*_x_* under alternating hydrogen and air atmospheres provides a dynamic sensing mechanism. PdH*_x_* forms rapidly in the presence of hydrogen and decomposes upon exposure to air, with hydrogen being released and oxidized [96]. This reversible process is closely associated with resistance variation, thereby contributing to improved repeatability and stability.In addition, PdH*_x_* formation facilitates hydrogen spillover toward the WO_3_ surface. Hydrogen atoms stored within Pd can diffuse to the interface and subsequently react with adsorbed oxygen species, releasing electrons back to the conduction band. This spillover-assisted process enhances both sensitivity and response speed. When PdO*_x_* is present, it can undergo partial reduction to metallic Pd under hydrogen exposure, enabling subsequent PdH*_x_* formation [98]. Therefore, PdO*_x_* functions both as an oxygen-active phase and as a precursor to metallic Pd. The dynamic interconversion between PdO*_x_* and Pd during sensing gives rise to a synergistic mechanism involving oxygen activation, hydrogen dissociation, hydride formation, and interfacial electronic modulation, ultimately leading to enhanced hydrogen sensing performance. The band alignment relationships between Pd/Pt/PdO/PtO and WO_3_ are illustrated in Figure 10a,d,j,g, respectively. The reason why the 2 at% Pt–2 at% Pd-decorated WO_3_ sensor exhibits the optimal sensing performance can be understood as a balance among catalytic sensitization, electronic sensitization, and surface accessibility. At relatively low Pt/Pd decorating contents, the number of catalytic active sites for H_2_ dissociation and oxygen activation is limited, and the amount of metal/semiconductor and oxide/semiconductor heterointerfaces is insufficient. As a result, the spillover effect and interfacial depletion-layer modulation are not fully developed. When the Pt and Pd contents are increased to 2 at%, abundant Pt/PtO and Pd/PdO*_x_* active sites are introduced onto the WO_3_ nanofibers, which promotes H_2_ dissociation, oxygen activation, hydrogen spillover, PdH*_x_*-related modulation, and interfacial charge transfer. These effects jointly amplify the resistance change upon exposure to H_2_ and reduce the optimal operating temperature.

However, further increasing the noble-metal content does not necessarily lead to continuous improvement. Excessive Pt/Pd decoration may partially cover the active WO_3_ surface, reduce the number of accessible oxygen adsorption sites, and hinder gas diffusion through the sensing layer. In addition, excessive noble-metal species may weaken the effective modulation of the WO_3_ depletion layer by forming overly dense or partially aggregated catalytic domains. Therefore, the 2 at% Pt–2 at% Pd composition provides the most favorable balance between sufficient catalytic/electronic sensitization and preservation of accessible WO_3_ surface sites, leading to the highest H_2_ response and the reduced operating temperature observed experimentally. In summary, the optimized 2 at% Pt–2 at% Pd decorating ratio enables a balanced contribution from Pt/PtO, Pd/PdO*_x_*, and WO_3_, thereby maximizing the synergistic effects of catalytic sensitization, electronic sensitization, hydrogen spillover, and depletion-layer modulation. This balance is responsible for the superior H_2_ sensing performance of Sensor B. Although the enhanced H_2_-sensing performance of Sensor B is attributed to the synergistic catalytic and electronic sensitization effects of Pt/Pd co-decoration, the direct verification of the spillover contribution requires further control experiments. In particular, a physical mixture of Pt-decorated WO_3_ and Pd-decorated WO_3_ nanofibers could serve as a useful reference to distinguish the effect of simple coexistence of Pt and Pd species from the interfacial synergy generated by Pt–Pd co-decoration on the same WO_3_ nanofiber surface. Such a control experiment would help clarify the contribution of spillover-related catalytic activation more directly and will be considered in our future work.

## 4. Conclusions

In this work, Pt/Pd co-decorated WO_3_ nanofibers were successfully prepared by electrospinning combined with calcination and investigated as sensing materials for ppb-level H_2_ detection. The optimized 2 at% Pt–2 at% Pd-decorated WO_3_ nanofibers, denoted as Sample B, exhibited the best gas-sensing performance among the prepared samples. The corresponding Sensor B showed a high response toward H_2_ at an optimized operating temperature of 170 °C, with reliable experimental detection down to 100 ppb H_2_. Based on the signal-to-noise criterion, the theoretical LOD was estimated to be approximately 1.4 ppb, further demonstrating the excellent trace-level H_2_ detection capability of Sensor B. In addition, Sensor B exhibited good selectivity against representative interfering gases, repeatable response–recovery behavior at 100 ppb H_2_ with an RSD below 1.0%, and long-term stability over 120 days.

The improved H_2_-sensing performance of Sensor B is mainly attributed to the synergistic effects of Pt/PtO, Pd/PdO*_x_*, and WO_3_. The noble-metal/metal-oxide interfaces can promote catalytic sensitization, electronic sensitization, hydrogen activation, possible spillover-related processes, and modulation of the surface depletion layer. In particular, the 2 at% Pt–2 at% Pd composition provides a favorable balance between sufficient noble-metal active sites and accessible WO_3_ surface sites, which is beneficial for enhancing the sensing response while maintaining efficient gas diffusion and surface reaction. Compared with representative Pt/Pd-modified WO_3_ hydrogen sensors reported in the literature, Sensor B shows clear competitiveness in terms of ppb-level detection, moderate operating temperature, response repeatability, and long-term operational stability.

Although Sensor B demonstrates promising H_2_-sensing performance, further efforts are still needed to improve its practical applicability. Future work will focus on optimizing noble-metal dispersion, introducing humidity calibration or compensation, and evaluating the sensor under more realistic hydrogen leakage environments. These efforts are expected to further improve the long-term reliability, environmental adaptability, and practical application potential of Pt/Pd co-decorated WO_3_ nanofiber-based hydrogen sensors.

## Figures and Tables

**Table 1:** A performance comparison between our sensor and other representative Pt/Pd-modified ${\mathrm{WO}}_{3}$ hydrogen sensors.

Materials	Temperature (°C)	H_2_ Concentration	Response	Response Time	Recovery Time	Detection Limit	Ref.
$\mathrm{PdO}-\mathrm{Pt}-{\mathrm{WO}}_{3}$ film	110	200 ppb	1.13	301 s	120 s	200 ppb	[49]
Pd−Pt alloy/${\mathrm{WO}}_{3}$ nanorods	RT	500 ppm	4	70 s	—	—	[48]
$\mathrm{Pd}-{\mathrm{WO}}_{3}$ nanoplates	200	100 ppm	1.67	8 s	10 s	100 ppm	[79]
$\mathrm{Pd}-{\mathrm{WO}}_{3}$ self-assembled	RT	100 ppm	1786.3	41 s	—	1 ppm	[78]
$\mathrm{Pt}-{\mathrm{WO}}_{3}$ nanorods	200	3000 ppm	2.2 × 10^5^	3.4 min	4.0 min	0.5 ppm	[80]
$\mathrm{PdO}/{\mathrm{WO}}_{3}$ nanospindles	150	50 ppm	76	1 s	214 s	1 ppm	[81]
$\mathrm{PdO}-{\mathrm{WO}}_{3}$ nanoneedles	200	500 ppm	1500	2 min	12 min	—	[82]
$\mathrm{Pd}-{\mathrm{WO}}_{3}$ nanoparticle	50	500 ppm	22,867	1.2 s	5–99 s	5 ppm	[83]
$\mathrm{Pt}/{\mathrm{WO}}_{3}$ nanotubes	450	500 ppm	17.6	25 s	—	10 ppm	[84]
$\mathrm{Pd}-\mathrm{decorated}\;{\mathrm{WO}}_{3}$ nanoigloos	200	50 ppm	115.28	9 s	—	—	[70]
$\mathrm{Pt}-\mathrm{Pd}-{\mathrm{WO}}_{3}$ nanofibers	170	100 ppb	1.57	70 s	100 s	100 ppb	This work

## Data Availability

The original contributions presented in this study are included in the article/Supplementary Materials. Further inquiries can be directed to the corresponding authors.

## Supplementary Materials

The following supporting information can be downloaded at: https://www.mdpi.com/article/10.3390/s26103079/s1, Figure S1: The XPS full spectrum of the Pt–Pd-decorated WO_3_ nanofibers sensing material; Figure S2: (a–e) Dynamic response curves of the sensor based on 1 at% Pd-decorated WO_3_ towards 100 ppm H_2_, measured at operating temperatures of 140 °C, 170 °C, 200 °C, 230 °C, and 260 °C, respectively, corresponding to Figure 5a in the text; Figure S3: (a–e) Dynamic response curves of the sensor based on 3 at% Pd-decorated WO_3_ towards 100 ppm H_2_, measured at operating temperatures of 140 °C, 170 °C, 200 °C, 230 °C, and 260 °C, respectively, corresponding to Figure 5a in the text; Figure S4: (a–e) Dynamic response curves of the sensor based on 2 at% Pd–1 at% Pt-decorated WO_3_ toward 100 ppm H_2_, measured at operating temperatures of 80 °C, 110 °C, 140 °C, 170 °C, and 200 °C, respectively, corresponding to Figure 5b in the text; Figure S5: (a–e) Dynamic response curves of the sensor based on 2 at% Pd–3 at% Pt-decorated WO_3_ toward 100 ppm H_2_, measured at operating temperatures of 80 °C, 110 °C, 140 °C, 170 °C, and 200 °C, respectively, corresponding to Figure 5b in the text; Figure S6: (a–d) The selectivity test of the sensor towards reference gas, corresponding to Figure 8d in the text; Figure S7: (a–f) The long-term stability test of the sensor, corresponding to Figure 8f in the text; Figure S8: (a–c) SEM images of the sensing material after coating onto the sensor device at different magnifications. The coated layer is composed of Pt/PtO and Pd/PdO*_x_* co-decorated WO_3_ nanofiber fragments and exhibits a porous fibrous morphology. (d) Cross-sectional SEM image of the coated sensing layer. Since it is difficult to obtain a completely uniform coating thickness, only a representative cross-sectional SEM image at the 200 μm scale is provided for thickness reference; Figure S9: EDS elemental mapping results of the Pt–Pd-decorated WO_3_ nanofibers. (a) Merged elemental mapping image of all detected elements; (b) Pd elemental mapping; (c) W elemental mapping; (d) Pt elemental mapping; (e) O elemental mapping; and (f) the corresponding SEM image.
