# Supplementary material for: A Highly Sensitive ppb-Level H2 Gas Sensor Based on Pt/PtO and Pd/PdOx Co-Decorated WO3 Nanofibers Prepared by Electrospinning
Source: Sensors (Basel). 2026 May 13;26(10):3079. doi: 10.3390/s26103079 (PMC13210745; doi:10.3390/s26103079)
Supplement: Supplementary file 1 [file sensors-26-03079-s001.zip › sensors-4284280-supplementary.pdf]

Supporting Information

A Highly Sensitive ppb-Level H_2 Gas Sensor Based on Pt/PtO and Pd/PdO_x Co-Decorated WO_3 Nanofibers Prepared by Electrospinning

Zhipeng Tang[1] , Jinshun Wang[1] , Lixin Zhang[1], Qiuxia Li[1], Chen Yang[1]， Yuhao Pang[1], Yingying Yang[1], Jingwei Chen[1]*, Qingkuan Meng[1]* and Qiang Jing

[1]Laboratory of Functional Molecules and Materials, School of Physics and Optoelectronic Engineering, Shandong University of Technology, 266 Xincun Xi Road, Zibo 255000, China.

*Corresponding author(s). E-mail(s):

chenjingwei@sdut.edu.cn; qkmeng@sdut.edu.cn

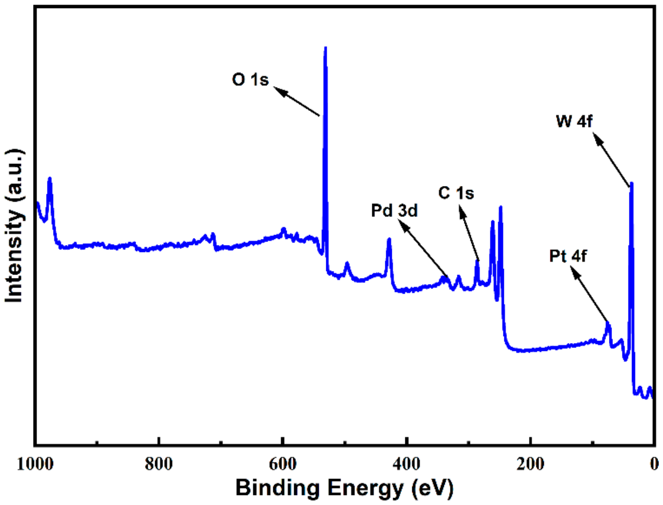

Fig. S1: The XPS full spectrum of the Pt－Pd-decorated WO_3 nanofibers sensing material.

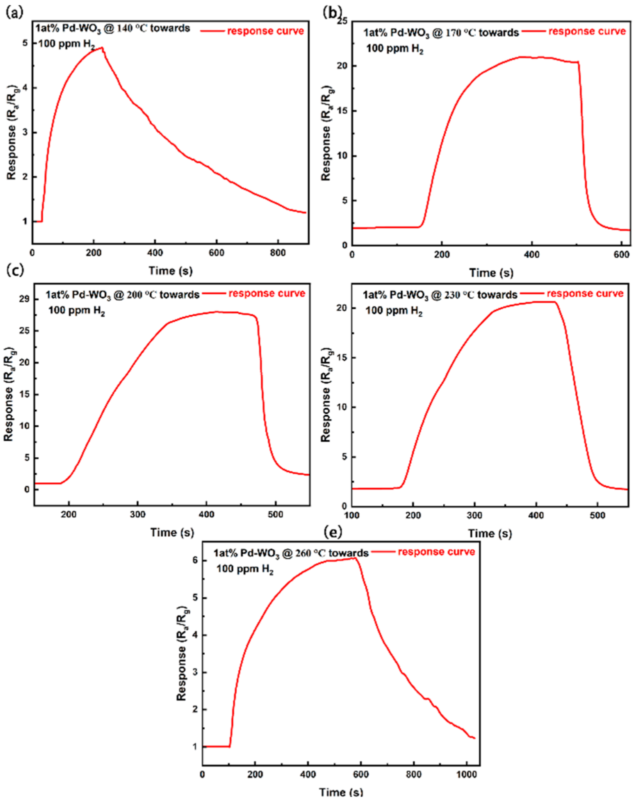

Fig. S2: (a)–(e) Dynamic response curves of the sensor based on 1 at% Pd-decorated WO_3 towards 100 ppm H_2, measured at operating temperatures of 140 ℃ 170 ℃, 200 ℃, 230 ℃, and 260 ℃, respectively, corresponding to Fig.5 (a) in the text.

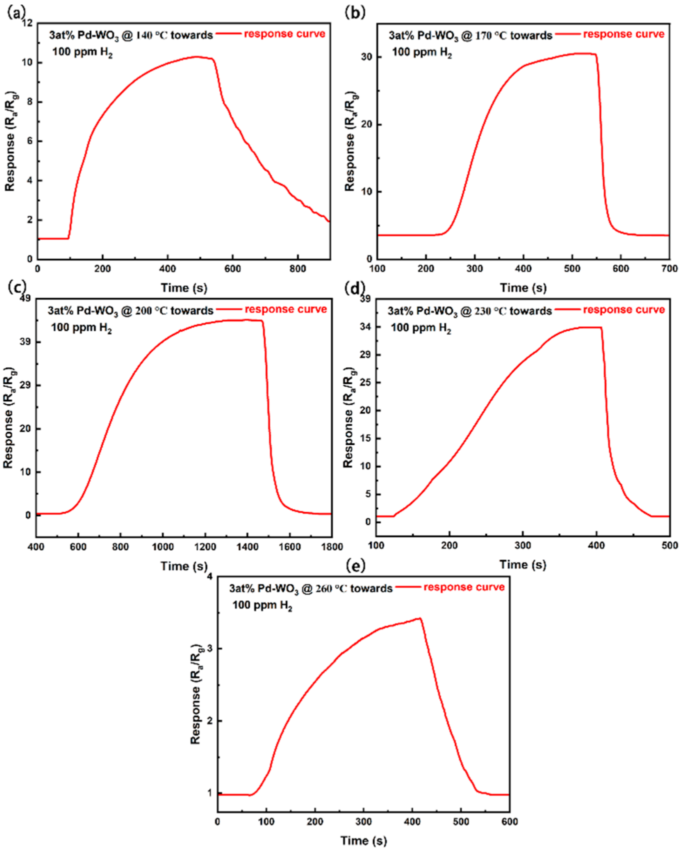

Fig. S3: (a)–(e) Dynamic response curves of the sensor based on 3 at% Pd-decorated WO_3 towards 100 ppm H_2, measured at operating temperatures of 140 ℃, 170 ℃, 200 ℃, 230 ℃, and 260 ℃, respectively, corresponding to Fig.5 (a) in the text.

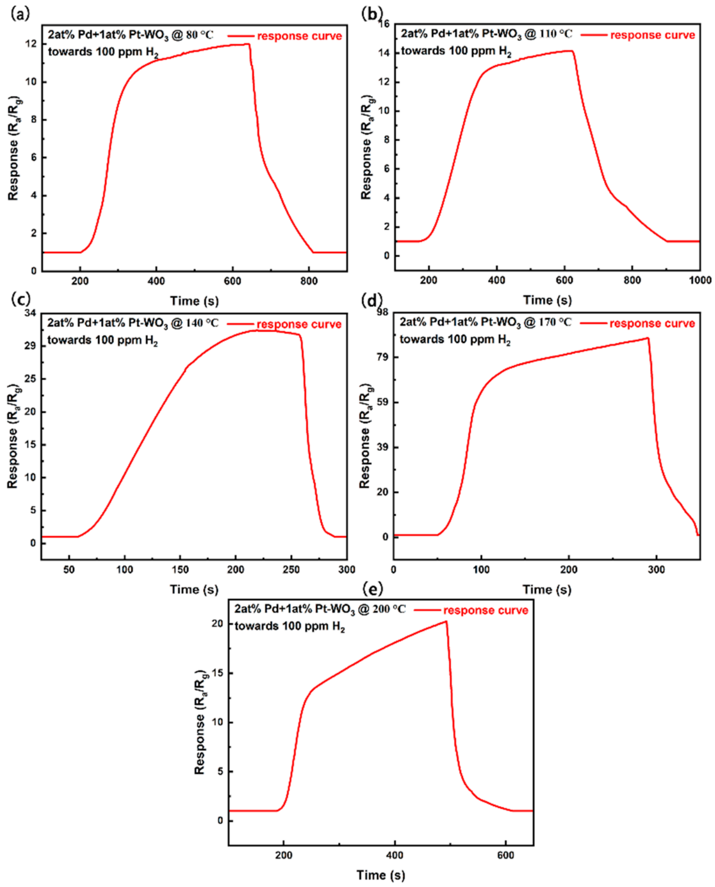

Fig. S4: (a)–(e) Dynamic response curves of the sensor based on 2 at% Pd–1 at% Pt-decorated WO_3 toward 100 ppm H_2, measured at operating temperatures of 80 ℃, 110 ℃, 140 ℃, 170 ℃, and 200 ℃, respectively, corresponding to Fig.5 (b) in the text.

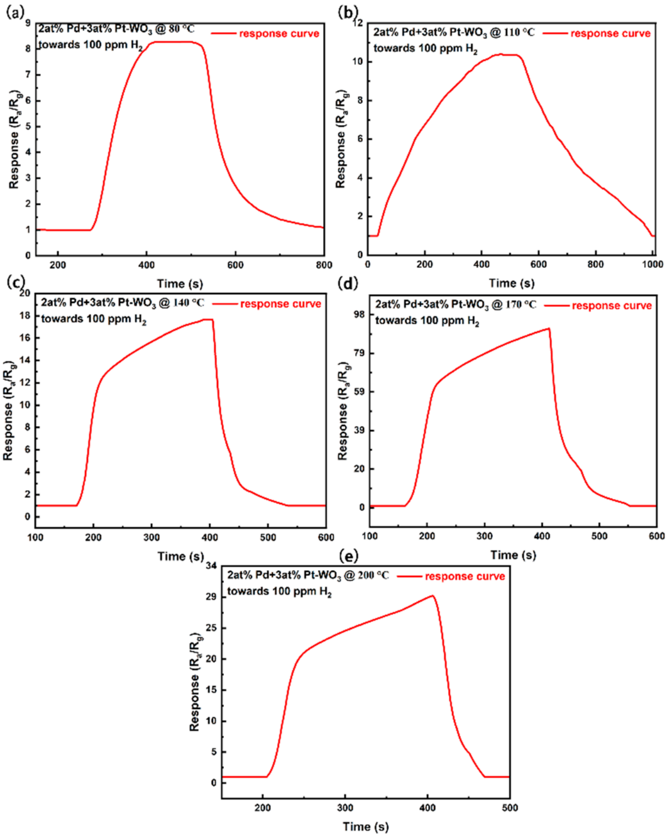

Fig. S5: (a)–(e) Dynamic response curves of the sensor based on 2 at% Pd–3 at% Pt-decorated WO_3 toward 100 ppm H_2, measured at operating temperatures of 80 ℃, 110 ℃, 140 ℃, 170 ℃, and 200 ℃, respectively, corresponding to Fig.5 (b) in the text.

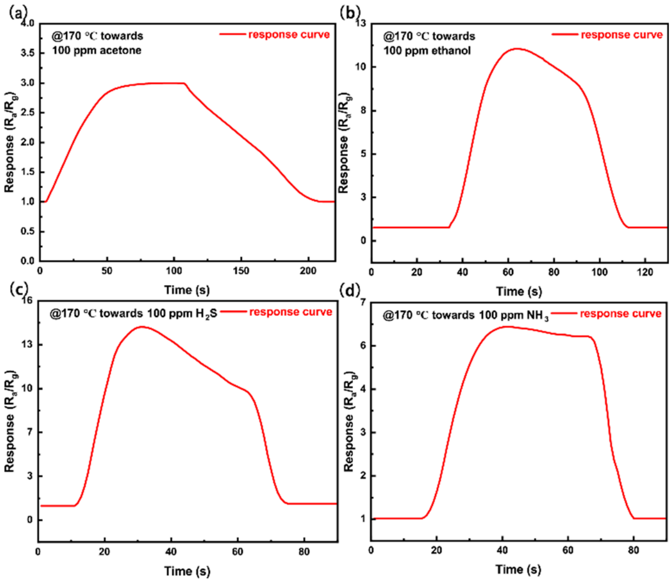

Fig. S6: (a)-(d) The selectivity test of the sensor towards reference gas, corresponding to Fig.8(d) in the text.

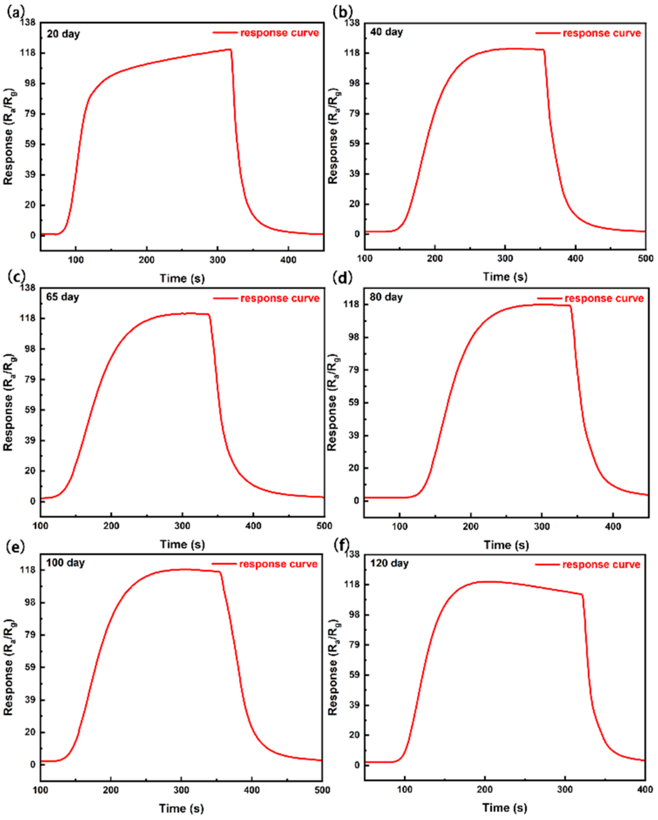

Fig. S7: (a)-(f) The long-term stability test of the sensor, corresponding to Fig.8(f) in the text.

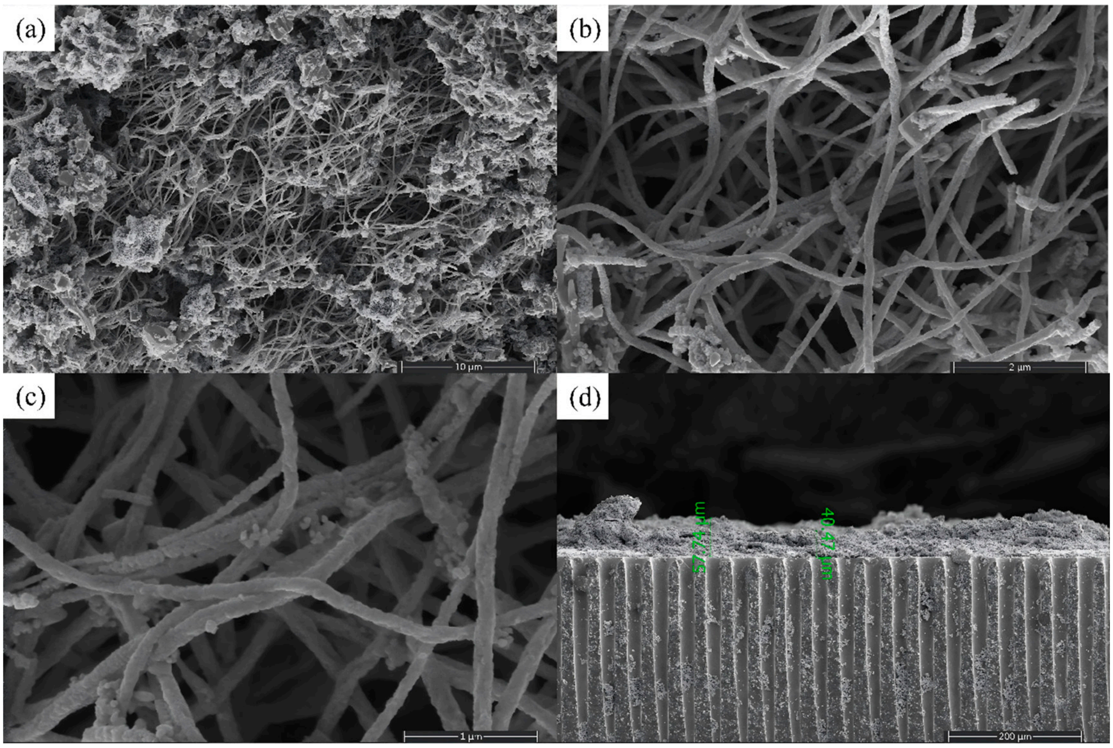

Fig. S8: (a–c) SEM images of the sensing material after coating onto the sensor device at different magnifications. The coated layer is composed of Pt/PtO and Pd/PdO_x co-decorated WO_3 nanofiber fragments and exhibits a porous fibrous morphology. (d) Cross-sectional SEM image of the coated sensing layer. Since it is difficult to obtain a completely uniform coating thickness, only a

representative cross-sectional SEM image at the 200 μm scale is provided for thickness reference.

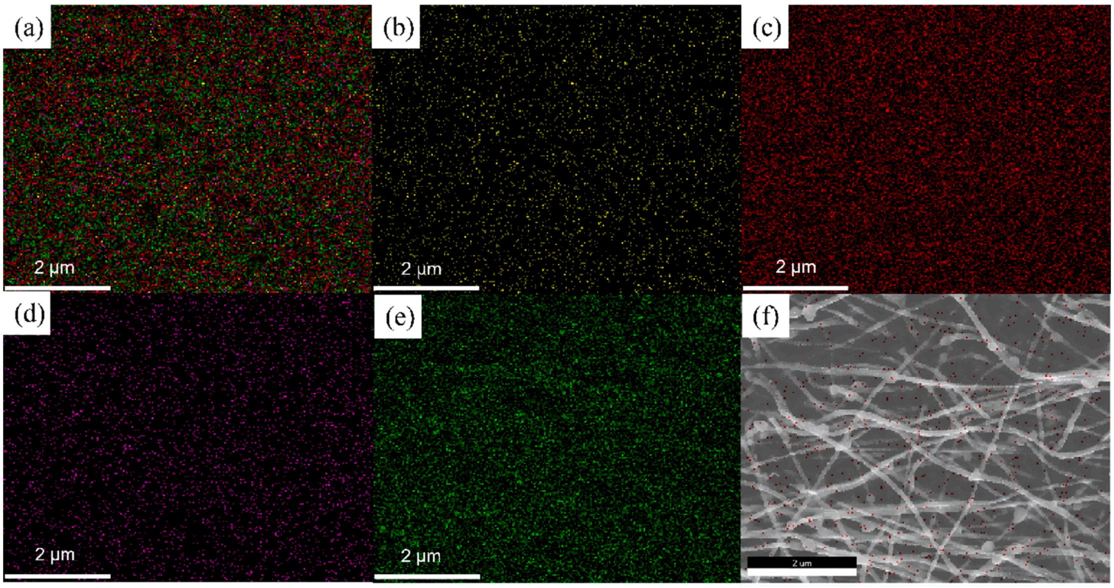

Figure S9: EDS elemental mapping results of the Pt–Pd-decorated WO_3 nanofibers. (a) Merged elemental mapping image of all detected elements; (b) Pd elemental mapping; (c) W elemental mapping; (d) Pt elemental mapping; (e) O elemental mapping; and (f) the corresponding SEM image.
